# Differentiation of the Organoleptic Volatile Organic Compound Profile of Three Edible Seaweeds

**DOI:** 10.3390/metabo13060713

**Published:** 2023-05-31

**Authors:** Pedro Catalão Moura, Jorge Manuel Fernandes, Mário Sousa Diniz, Viktor Fetter, Valentina Vassilenko

**Affiliations:** 1 Laboratory for Instrumentation, Biomedical Engineering and Radiation Physics (LIBPhys—UNL), Department of Physics, NOVA School of Science and Technology, NOVA University of Lisbon, Campus FCT-UNL, 2829-516 Caparica, Portugal; 2 NMT, S. A., Edifício Madan Parque, Rua dos Inventores, 2825-182 Caparica, Portugal; 3 Applied Molecular Biosciences Unit (UCIBIO), Department of Chemistry, NOVA School of Science and Technology, NOVA University of Lisbon, 2829-516 Caparica, Portugal; 4 Airbus Defense and Space GmbH, Space Systems, Department of TESXS Science Engineering, 88046 Friedrichshafen, Germany

**Keywords:** edible seaweeds, grateloupia turuturu, codium tomentosum, bifurcaria bifurcata, ion mobility spectrometry, gas chromatography, volatile organic compounds

## Abstract

The inclusion of seaweeds in daily-consumption food is a worthy-of-attention challenge due to their high nutritional value and potential health benefits. In this way, their composition, organoleptic profile, and toxicity must be assessed. This work focuses on studying the volatile organic compounds (VOCs) emitted by three edible seaweeds, *Grateloupia turuturu*, *Codium tomentosum*, and *Bifurcaria bifurcata*, with the aim of deepening the knowledge regarding their organoleptic profiles. Nine samples of each seaweed were prepared in glass vials, and the emitted headspace was analyzed, for the first time, with a gas chromatography—ion mobility spectrometry device, a highly sensitive technology. By statistically processing the collected data through PCA, it was possible to accurately differentiate the characteristic patterns of the three seaweeds with a total explained variance of 98%. If the data were pre-processed through PLS Regression, the total explained variance increased to 99.36%. The identification of 13 VOCs was accomplished through a developed database of compounds. These outstanding values in addition to the identification of the main emissions of VOCs and the utilization of a never-before-used technology prove the capacity of GC-IMS to differentiate edible seaweeds based solely on their volatile emissions, increase the knowledge regarding their organoleptic profiles, and provide an important step forward in the inclusion of these highly nutritional ingredients in the human diet.

## 1. Introduction

The inclusion of seaweeds in the diet and foods of daily consumption is a trending and worthy-of-attention challenge due to their high nutritional value and potential benefits. In opposition to Eastern societies, where the consumption of seaweeds is an ordinary and common practice, Western societies have just recently approved legislation regarding the human consumption of algae-based products [1,2]. In this way, the full characterization of seaweeds regarding their composition, nutritional relevance, organoleptic profiles, or toxicity, among other topics, is a mandatory but demanding topic.

Algae are uni- or multicellular organisms that live in water or locations with elevated levels of humidity. Unicellular seaweeds are often categorized as microalgae, which are commonly known as phytoplankton and cannot be seen by the naked eye. On the other hand, multicellular algae belong to the category of macroalgae, can be seen without a microscope, and are commonly addressed as seaweeds. They are mostly autotrophic, and chlorophyll is their primary photosynthetic pigment. Nonetheless, other pigments existent in their structure can equally influence the color of the seaweed [3,4].

The color of seaweed is commonly used to group these organisms. Three main groups are often considered: green seaweeds, red seaweeds, and brown seaweeds. The green pigmentation of the green algae, which belong to the phylum *Chlorophyta*, is due to the abundant presence of chlorophyll in their composition. Red seaweeds belong to the phylum *Rhodophyta*, and their color is directly related to some photosynthetic pigments that play an accessory role to chlorophyll, namely phycobiliproteins, such as phycoerythrin or phycocyanin. Included in the phylum *Ochrophyta*, brown algae exhibit their darker coloration due to the existence of a specific group of pigments in their structure, the carotenoids [4,5].

Independently of the category, both microscopic and macroscopic algae represent an abundant source of biological assets, such as protein, dietary fiber, vitamins, fatty acids, carbohydrates, and minerals [6]. In this way, seaweeds have been exploited for several scientific and civil fields, such as pharmaceutical and biomedical industries, alternative medicine, cosmetics, agriculture, and, in some societies, as food ingredients [7,8]. As previously addressed, their application in food is still a demanding task, especially in Western societies. Even considering the well-known antioxidant, anti-viral, anti-microbial, anti-aging, and anti-carcinogenic properties of the seaweeds, further scientific studies regarding the composition, organoleptic profile (flavor, odor, or texture), nutritional value, emissions, possible hazardousness, and toxicity of these organisms are required before their full integration into the diet of the society [4,5].

The organoleptic characteristics of seaweeds are directly related to the volatile compounds present in their structure, namely ketones, aldehydes, aliphatic and aromatic hydrocarbons, halogenated compounds, and sulfur-based compounds, among many others [9,10]. Nonetheless, not all the volatile compounds present in algae are equally important to odor and flavor, and the degree of contribution to the organoleptic properties hinges on their recognition threshold and concentration [11,12]. Although aliphatic hydrocarbons constitute the majority of volatile organic compounds (VOCs) released by seaweeds, their contribution to the aroma profile is almost zero [13]. Odor-related compounds are derived, in great part, from polyunsaturated fatty acids. However, most of the formation mechanisms of these volatile compounds, such as the presence of halogenated sesquiterpenes, diterpenes, and acetylenes in red algae, are still not fully understood [14,15]. Despite this, VOCs are still the main analytes responsible for the organoleptic profiles of seaweeds and deserve proper attention, not only due to this fact but also due to their potential hazardousness and toxicity for humans [16,17].

Considering the importance of VOCs regarding the odor and flavor of seaweeds, the assessment of the spoilage levels, and human health, this study intends to, for the first time ever, apply a GC-IMS device to fully characterize the organoleptic profile and, specifically, the VOCs emitted by three edible seaweeds, *Grateloupia turuturu* (GT, red seaweed of the phylum *Rhodophyta*), *Codium tomentosum* (CT, green seaweed of the phylum *Chlorophyta*), and *Bifurcaria bifurcata* (BB, brown seaweed of the phylum *Ochrophyta*) and, consequently, deepen the knowledge regarding these very valuable sources of biological assets and medically relevant organisms.

As mentioned, a GC-IMS device was, for the first time ever, used for the seaweed analyses. IMS is a widely-used analytical technology with elevated levels of sensitivity, high selectivity, instrumental simplicity, and analytical flexibility, and it is capable of separating and identifying a vast range of ionized molecules of a volatile sample in concentration ranges as low as low ppb_v_ and ppt_v_ [18,19,20]. Furthermore, IMS requires a short range of time for sample analysis, providing results in quasi-real time [21,22]. When coupled with gas chromatography (GC), the resulting device presents increased levels of sensitivity and selectivity that enable the separation and analysis of complex matrices rich in VOCs, even at trace concentration levels [23,24,25]. For these reasons, this device presents a promising future for the scientific field of organoleptic VOC profiling of edible macroalgae.

## 2. Materials and Methods

### 2.1. Seaweeds Samples

Three edible seaweeds, one of each photosynthetic pigment-based category, were considered for this study: *Grateloupia turuturu* (GT), *Codium tomentosum* (CT), and *Bifurcaria bifurcata* (BB). Known as Devil’s tongue weed, *Grateloupia turuturu* is a red macroalga from the phylum *Rhodophyta*, which presents a gelatinous texture and whose leaves can grow as long as 70 cm and as wide as 15 cm. Common in water bodies of Asian countries, its presence in Europe and America is considered invasive. *Codium tomentosum* is a macroalga of the phylum *Chlorophyta*. This seaweed is commonly known as Sponge weed due to its spongy texture and is native to the Atlantic coast of Europe and parts of Africa. Its branched structure can extend up to 30 cm long. Finally, the brown macroalgae studied during this study, *Bifurcaria bifurcata*, belongs to the phylum *Ochrophyta* and has the name of Brown forking weed. This seaweed is commonly found on European and American sea shores, specifically in rock-rich coastal zones. BB presents a branched structure that can grow up to 30 cm long, like CT. All the samples of seaweeds were collected during the spring of 2019 at Figueira da Foz on the Portuguese coast. CT and BB were collected at Cabo Mondego, and GT was collected at Buarcos. Once collected, the samples were washed and stored at −15 °C.

### 2.2. Sample Preparation

**Figure 1:**
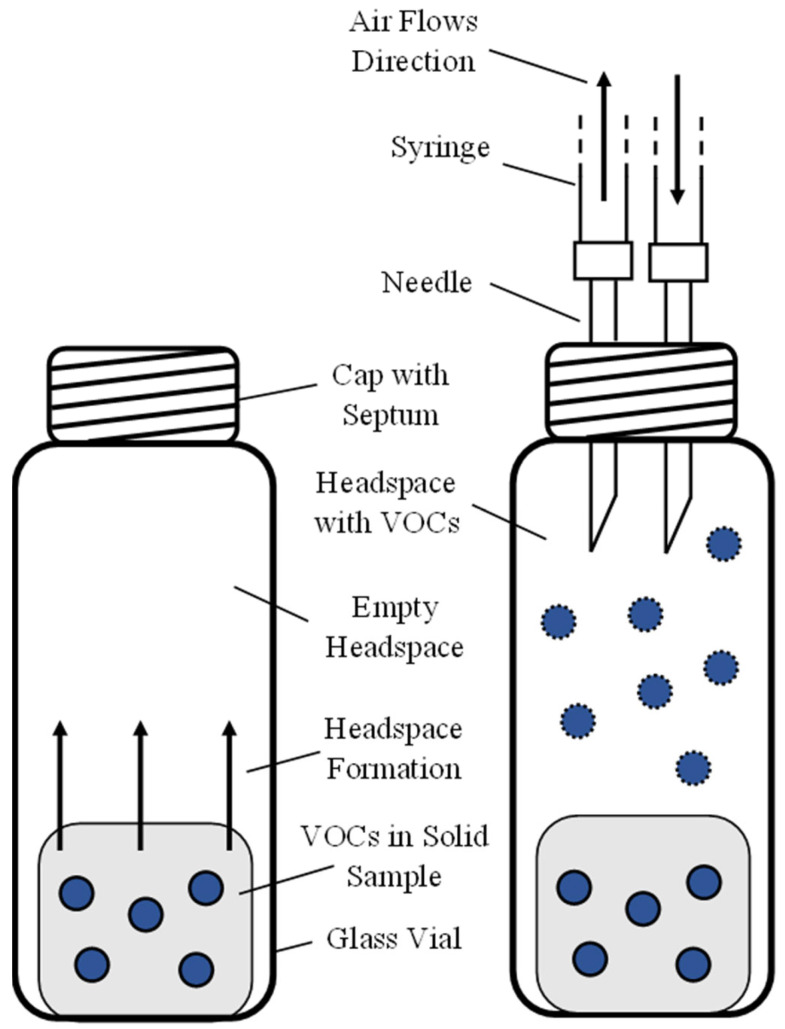
Schematic of the headspace formation and collection. A solid sample rich in VOCs is placed in the interior of a glass vial (**left**), and once the headspace is formed, it is collected with the help of two needles (**right**).

A total of 9 samples per species of seaweed were prepared and analyzed with the spectrometer. To do so, 0.5 g of alga was placed in the interior of a 20 mL sterile glass vial. Then, the vial was closed with a spectrum cap, isolating the samples from potential contamination or exogenous compounds. After the preparation, the samples were left to create headspace at room temperature. Once the thermodynamic balance between the solid and the volatile sections of the sample was reached, a 2 mL portion of the headspace was collected with a sterile syringe and injected into the GC-IMS device. Figure 1 schematizes the procedure utilized for collecting the sample from the vial.

It is worth noting that the quality control of the method was assured by the consecutive measurement of the nine replicates for each seaweed as well as by the measurements of the blank spectra before each experimental run of algae samples. The measurement of nine replicates ensured the statistical relevance of the data collected and the overall legitimacy of the results. For the measurement of the blank samples, headspace samples from virgin vials without any sample besides room air were analyzed with the spectrometer in a similar procedure to that described before. In this way, the measurement of the blank samples ensured the accurate assessment of the pattern of VOCs for each seaweed analyzed during the study by providing information on the content of the room air.

### 2.3. Instrumentation

For the first time ever, a GC-IMS device was used to assess the organoleptic profile of the VOCs emitted by edible seaweeds. A GC-IMS analysis starts with the injection of the volatile sample into the spectrometer. Here, the sample suffers pre-separation in the interior of the chromatographic column, i.e., the analytes are separated based on their capacity to adsorb to the inner coating of the column. Each analyte requires a specific time to elute from the column. This time is commonly defined as the retention time, r_t_, and, due to being compound-specific, it allows the identification of all the VOCs existent in the original sample [26,27].

Once eluted from the GC, the analytes pass to the IMS section of the device. Here, they are ionized by an ionization source. After ionization, the newly formed ions are exposed to a weak and homogeneous electric field that will lead the ions to drift along the entire drift tube of the spectrometer. Here, they are detected at a specific time known as the drift time, d_t_. Further details regarding the GC-IMS operating principle can be consulted elsewhere [24,27]. Nonetheless, it is important to state that the detected ions are assessed considering their ion mobility constants.

The ion mobility constant, usually represented by K, is a compound-specific value that is calculated considering the drift velocity, v_d_, of the ions drifting throughout the tube and the intensity of the electric field, E, mentioned previously. The drift velocity of each ion is also a specific value that depends on the length of the tube, L, and the characteristic drift time, d_t_, of the target ion [28,29,30,31]. In this way, K can be represented as (Equation (1)):

(1) $$K=\frac{v_{d}}{E}=\frac{L}{E.t_{d}}$$

Despite being compound-specific values, ion mobility constants are dependent on the pressure and temperature conditions during the analysis. To overcome this issue, they are typically normalized to standard environmental levels of pressure (P_0_ = 760 Torr) and temperature (T_0_ = 273.15 K). The normalized ion mobility constant is represented by K_0_, and it can be represented as (Equation (2)) [28,29,30].

(2) $$K_{0}=K.\frac{P}{P_{0}}.\frac{T_{0}}{T}$$

**Figure 2:**
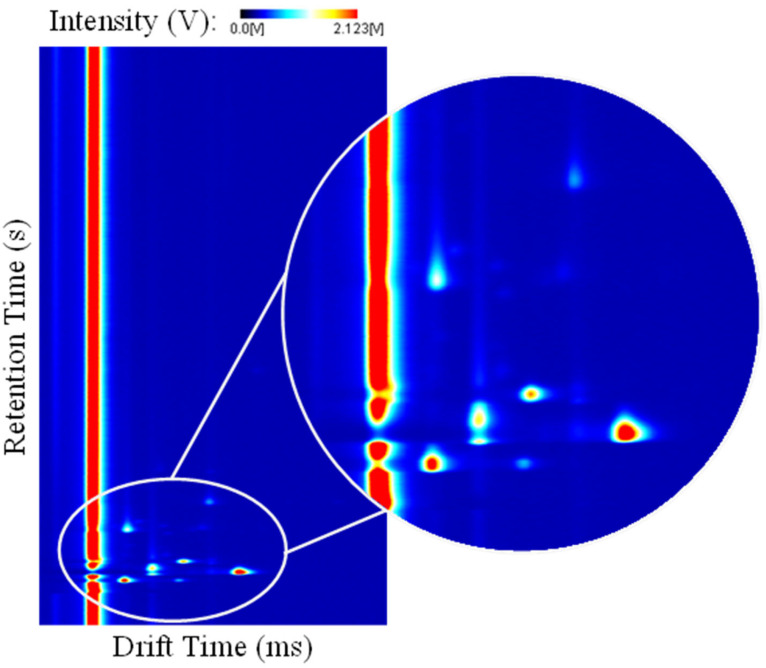
Three-dimensional gas chromatography—ion mobility spectrometry spectrum. Here, the drift time (ms) and retention time (s) are represented in the x- and y-axes, respectively. The intensity is represented with a color map. An enlarged section was added for visualization purposes.

Once the measurement is concluded, a three-dimensional spectrum is produced. Here, three variables are presented. The x- and y-axes usually represent the drift (milliseconds) and retention (seconds) times, respectively, registered during the analysis. A third coordinate, corresponding to the intensity (volts) of each ion detected by the detector placed at the end of the drift tube, is equally represented. These intensity levels are directly related to the original concentrations that each molecule presented in the original sample and can be used for plotting calibration curves and quantifying the detected analytes [32]. Figure 2 schematizes an arbitrary three-dimensional GC-IMS spectrum in a two-dimensional view. Here, the drift and retention times are represented in the x- and y-axis, respectively, and the intensity is represented through a color map. An enlarged section was added for visualization purposes.

As mentioned previously, the drift time, the drift velocity, the ion mobility constant, and the normalized ion mobility constant are analyte-specific values, so all of them allow one to accurately and safely identify all the VOCs detected during GC-IMS analysis. The intensity, in turn, can be used for quantification purposes, i.e., to assess the concentration of the detected analytes in the original sample.

The device used for algae analysis during this study was a GC-IMS apparatus from G.A.S. Dortmund (Germany), equipped with an MXT-200 chromatographic column with a length of 30 m and an internal diameter of 0.53 mm, which was coated with stainless steel with a mid-polar stationary phase of trifluoropropylmethyl polysiloxane with a thickness of 1 μm. As an ionization source, a Tritium source, 3H (β-radiation: 300 MBq), was assembled in the apparatus. The drift tube, with a length of 98 mm, presented a 5 kV switchable polarity and an electric field strength of 500 V/cm. The IMS operated at room temperature. Purified air was used as the drift and carrier gases. Further details regarding the GC-IMS operation parameters can be found in Table 1.

### 2.4. Data Processing

Once each analysis with the spectrometer concluded, the registered data, i.e., the drift and retention times and the intensity, were exported and processed using LAV software (GAS Dortmund—version 2.2.1). 

The intensity levels of all the analytes detected for a single sample of seaweed were studied to assess the repeatability of the data collected and, as mentioned previously, verify the suitability of the device. In this way, these values were statistically processed with a partial least squares (PLS) regression and by principal component analysis (PCA). All the statistical analyses were conducted with SPSS Statistics software (IBM—version 23).

The drift and retention times, in turn, were used for identification purposes. As addressed previously, both the retention and drift times are compound-specific values. In this way, they can be used to accurately identify specific analytes among all the detected ones, even in extremely complex matrices. To do so, a database of VOCs was internally developed by our research group. By crosschecking the drift and retention times of the compounds registered in the library of VOCs with the times collected during the analyses of the algae, it was possible to accurately identify the analytes emitted by the three edible algae.

To develop the library of VOCs, pure samples (20 µL) of the target analyte were prepared in 20 mL glass vials. Then, 2 mL of the created headspace was collected with a syringe and injected into the spectrometer. Once analyzed, the registered drift and retention times were exported and included in the database. The ion mobility and the normal ion mobility constants were calculated and equally registered in the database. At the time of this work, the database included information on 270 volatile organic compounds.

## 3. Results and Discussion

### 3.1. Seaweed Emission Patterns

**Figure 3:**
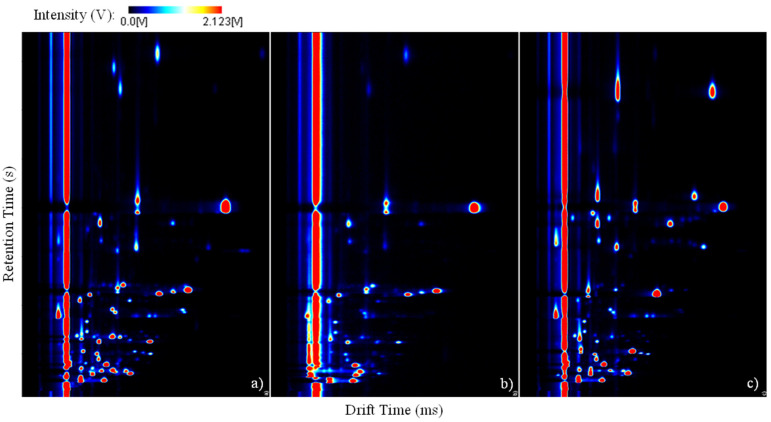
Three-dimensional spectra registered considering the volatile organic compounds emitted by the seaweeds: (**a**) *Grateloupia turuturu*, (**b**) *Codium tomentosum*, and (**c**) *Bifurcaria bifurcata*.

A total of nine analyses were performed for each one of the three edible seaweeds to ensure the statistical relevancy of the data and the quality of the results, resulting in a total of 27 three-dimensional spectra. Each seaweed presents a distinct composition. In this way, a characteristic pattern of the emission of VOCs can be expected. Figure 3 illustrates, in a two-dimensional view, one GC-IMS spectrum for each seaweed: (a) *Grateloupia turuturu*, (b) *Codium tomentosum*, and (c) *Bifurcaria bifurcata*. It is worth clarifying that the drift time is represented on the x-axis, and the retention time is represented on the y-axis. The intensity levels are represented by a color map. The long red bar visible throughout the entire spectrum corresponds to the reactant ion peak (RIP) of the GC-IMS device, and each peak visible in the spectra corresponds to the monomers, dimers, and even trimers of specific volatile organic compounds.

Each seaweed revealed a characteristic emission pattern that relates to its respective constitution. So, by visually analyzing the spectra and comparing the presence of the peaks, a unique fingerprint can be established for each seaweed. Comparable patterns are visible for the red and green seaweeds. However, in the 100–150 s range of the retention time, a higher incidence of analytes is visible in the spectrum of the green seaweed. On the other hand, the red seaweed seems to have at least two characteristic peaks in the range of 350–400 s of retention time that are absent from the spectrum of the green seaweed. The pattern of the brown seaweed exhibits more differences when compared with the remaining two. In fact, more intense peaks are visible in this seaweed, and two very characteristic and exclusive peaks are observable at around 350 s of retention time. Evidently, peaks common to all three seaweeds are discernible. However, in order to fully assess their true identity, an accurate identification has to be achieved.

### 3.2. Seaweed Differentiation

From the pattern of VOCs detected for all three seaweeds, a total of 105 independent peaks were observed. The third variable of these peaks, i.e., the intensity, was used to assess the repeatability of the data collected with the GC-IMS throughout the entire project and, consequently, evaluate the suitability of this technology to study the organoleptic profile of macroalgae.

**Figure 4:**
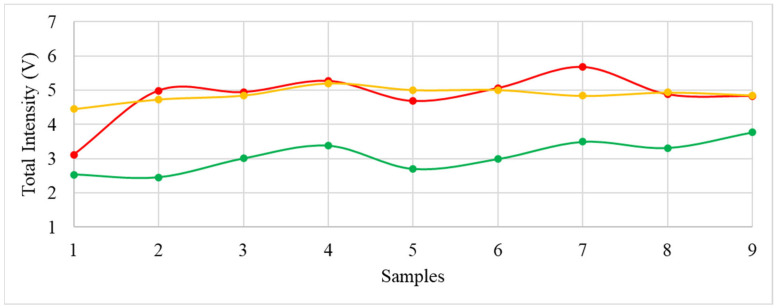
Variability of the total intensity detected with the GC-IMS device for the seaweeds *Grateloupia turuturu* (red line), *Codium tomentosum* (green line), and *Bifurcaria bifurcate* (orange line).

As the first approach, the intensity levels of all the analytes detected in each one of the nine analyses were summed, and variability graphs were plotted to assess the repeatability of the data. Figure 4 represents the variability of the total intensity registered by the GC-IMSA during the nine analyses performed for each seaweed. The variability of the samples for *Grateloupia turuturu*, *Codium tomentosum*, and *Bifurcaria bifurcata* are represented in red, green, and brown, respectively, to match the pigmentation of the seaweeds.

By visually inspecting the variability lines, it is possible to conclude that GC-IMS ensures the repeatability of the collected data throughout all the replicas analyzed for the same sample of seaweed. In fact, considering the total intensity registered for the nine replicas of the three seaweeds, mean and standard deviation values of (5.1 ± 0.5) V, (2.8 ± 0.6) V, and (4.8 ± 0.2) V were achieved for the red, green, and brown seaweeds, respectively, proving the desired repeatability and, consequently, the appropriateness of GC-IMS for this type of study.

**Figure 5:**
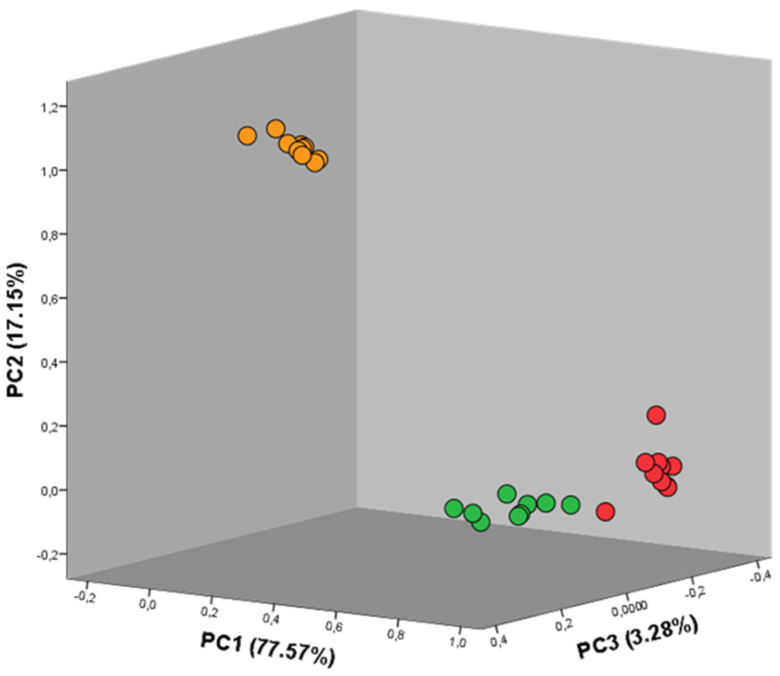
Principal component analysis graph for the three seaweeds. The circles exhibit the color of the corresponding seaweed, i.e., red circles: *Grateloupia turuturu*, green circles: *Codium tomentosum*, and orange circles: *Bifurcaria bifurcate*. A total explained variance of 98.00% was achieved.

Despite the variability in the results, it is scientifically important to assess whether the three considered seaweeds are fully differentiated based solely on the patterns of VOCs detected with the GC-IMS device. To do so, the total intensity levels of the 105 detected peaks were statistically processed through principal component analysis. Figure 5 illustrates the three principal components achieved during the PCA.

**Figure 6:**
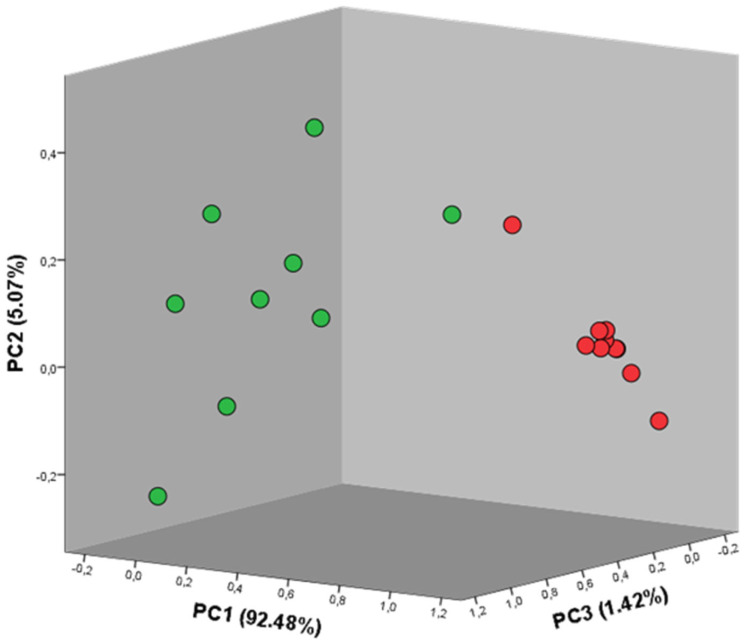
Principal component analysis graph for the red and green seaweeds. The circles exhibit the color of the corresponding seaweed, i.e., red circles: *Grateloupia turuturu* and green circles: *Codium tomentosum*. A total explained variance of 98.97% was achieved.

As represented in the axes, the explained variance calculated for the components PC1, PC2, and PC3 was 77.57%, 17.15%, and 3.28%, respectively. In this way, the three seaweeds were differentiated with a total explained variance of 98.00%. Each seaweed’s set of values is marked in the respective color (red, green, and brown), which makes the distinction between the three groups of values and, consequently, between the three seaweed species undoubtedly visible. Furthermore, a higher degree of separation is visible between the brown seaweed, BB, and both the red and green seaweeds whose differentiation is not as evident as desired. A second PCA was then developed in order to assess the differentiation between the algae *Grateloupia turuturu* and *Codium tomentosum*. Figure 6 illustrates this new set of components.

As previously mentioned, for this second PCA the intensity levels of the peaks detected in the patterns of the red and green seaweeds were just considered. As included in the axes, the components presented explained a variance of 92.48%, 5.07%, and 1.42%, respectively, which resulted in a total explained variance of 98.97%.

Considering the results of the PCA, it is safe to state that the BB seaweed possesses a more distinctive composition and, consequently, a different organoleptic profile when compared with the GT and CT seaweeds—a fact that is visible not only in the three-dimensional spectra but also in the graph of the principal components. When comparing the organoleptic profiles detected by the GC-IMS exclusively for the red and green seaweeds, the differentiation of both seaweeds is equally evident and scientifically solid.

**Figure 7:**
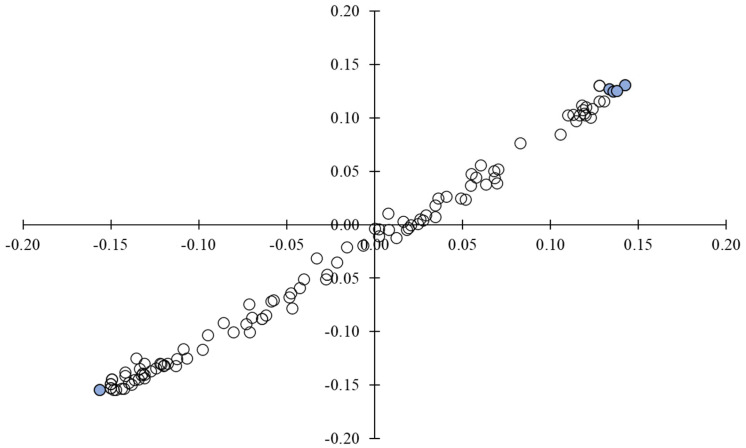
Graph of the partial least squares regression. The points selected for posterior processing are marked in blue.

These results reinforce the suitability of GC-IMS and the scientific validity of the collected data regarding the VOC profiles. Nonetheless, PCA is a dimension reduction technique that grants the conversion of correlated variables into a new uncorrelated set of values, but it does not take into consideration the correlation between independent and dependent variables. As a consequence of this feature, another dimension reduction technique was applied before the PCA: PLS regression. Partial least squares regression is a statistical method that finds a linear regression model that represents the best relation between the variable values and enables the reduction of the predictor number to a smaller group of components that are not correlated, considering the correlation among the independent and the dependent variables. The linear regression obtained by applying a PLS regression to the full data is shown in Figure 7. The values selected to plot the new PCA graph were selected based on the fact that they were the most distant points from the graph’s origin, which indicates less correlated values and, consequently, they were adequate to perform further analysis. Five values found by PLS regressions were used for the subsequent PCA.

**Figure 8:**
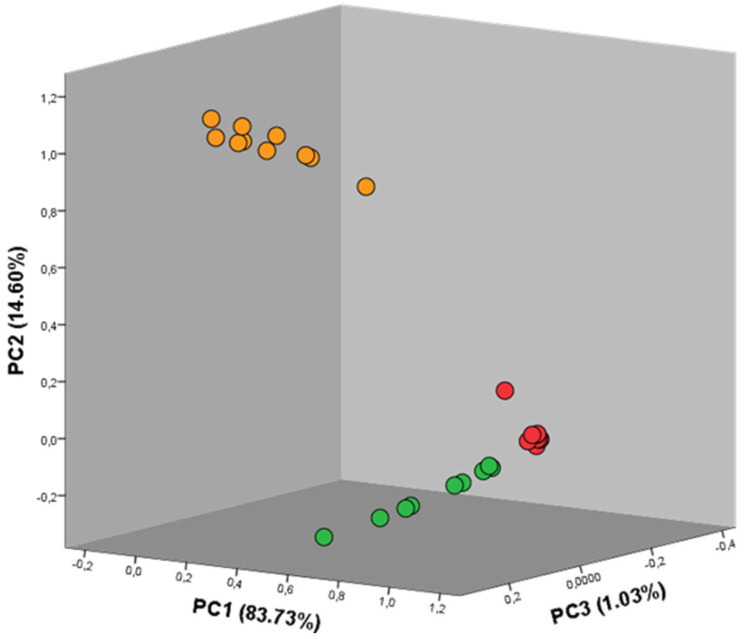
Principal component analysis graph for the three seaweeds. The circles exhibit the color of the corresponding seaweed, i.e., red circles: *Grateloupia turuturu*, green circles: *Codium tomentosum*, and orange circles: *Bifurcaria bifurcate*. A total explained variance of 99.36% was achieved.

A new PCA graph was then plotted considering solely the values selected during the PLS regression analysis. Figure 8 illustrates the new separation pattern and the respective components.

As can be seen in the figure, the principal components PC1, PC2, and PC3 increased their respective values of explained variance to 83.73%, 14.60%, and 1.03%. The differentiation between the three edible seaweeds considered during this study was, in this way, achieved with a total explained variance of 99.36%. Considering all the results achieved during the statistical analyses, it is safe to say that GC-IMS is capable of collecting repeatable and scientifically relevant data, and the differentiation of the organoleptic profiles from the *Grateloupia turuturu*, *Codium tomentosum*, and *Bifurcaria bifurcata* seaweeds, exclusively considering the volatile organic compounds emitted by the samples, is achievable with levels of confidence close to 100%.

### 3.3. VOC Identification

As previously addressed, this paper intends to give a step forward in the characterization of the organoleptic profile of three edible seaweeds based on their VOCs. To do so, the main VOCs emitted by the algae were identified considering a previously developed database. Among the monomers, dimers, and even some trimers, it was possible to identify a total of 13 volatile organic compounds, namely, 12 VOCs in the emission patterns of *Grateloupia turuturu*, 7 VOCs in the emissions of *Codium tomentosum*, and 8 analytes emitted by *Bifurcaria bifurcata*. Table 2 summarizes the VOCs identified per analyzed sample of seaweed.

To achieve the identification of these analytes, the specific drift and retention times of the analytes were used. These values can be found in Table 3.

All the listed VOCs were successfully detected in the emissions of the three edible seaweeds with the GC-IMS apparatus and accurately identified with the previously developed database. It is safe to state that, considering the patterns of the VOCs measured for each seaweed, their differentiation through principal component analysis and their characterization through the identification of the main analytes represents a step forward in the full characterization of the analyzed seaweeds, deepens the knowledge regarding their organoleptic profiles, and, overall, is a contribution to phycology.

## 4. Conclusions

The present study intended to study the organoleptic profile of three edible seaweeds and deepen our knowledge regarding their suitability for being included in food products and the overall Western diet. In this way, the volatile organic compounds emitted by three edible seaweeds, *Grateloupia turuturu*, *Codium tomentosum*, and *Bifurcaria bifurcata*, were analyzed for the first time ever with a gas chromatography—ion mobility spectrometry device.

Considering the three-dimensional spectra achieved during the analyses of nine samples of each seaweed, it was possible to verify the evident differences between the compositions of the algae regarding the volatile organic compounds existent in their structure and emitted to the atmosphere. The GC-IMS spectra revealed a unique and characteristic fingerprint for each seaweed. The red and green seaweed spectra unveiled similar patterns, while the brown seaweed fingerprint was evidently distinct. The repeatability of the spectra for several samples of the same seaweed with GC-IMS was undoubtedly proven. Additionally, it was possible to deepen the knowledge regarding those same VOCs through the identification of 14 specific analytes emitted by the algae. To do so, a database of analytes was previously developed, ensuring the scientific solidness of the results. The intensity levels of all the detected VOCs enabled the differentiation of all three algae, with a total explained variance of 98.00% through PCA. In addition, when previously processed with PLS, the total explained variance increased to 99.36%, which is an astonishing value that leaves no room to question the results.

The features of gas chromatography—ion mobility spectrometry, namely high sensitivity and selectivity, analytical flexibility, a low operating cost, easy operationality, and portability, proved to be more than suitable to completely study the samples of seaweeds and fully characterize the complex matrices of the VOCs emitted by those species. In this way, it is possible to predict an auspicious future for this technology in the characterization of new ingredients, the development of innovative food products, and in the world of phycology.

## Figures and Tables

**Table 1:** Operation parameters of the gas chromatography—ion mobility spectrometry device.

Parameters	Values	Units
Sample Loop Volume	1	mL
GC Column Model	MXT-200	–
GC Column Length	30	m
GC Column Diameter	0.53	mm
GC Temperature	343.15	K
Ionization Source	Tritium—β Radiation	–
Ionization Polarity	Positive	–
Drift Region Length	9.8	cm
Drift Potential Difference	5	kV
IMS Temperature	343.15	K
IMS Pressure Range	742–760	Torr
Gas Nature	Purified Air	–
Carrier Gas Flow	10–50	mL/min
Drift Gas Flow	150	mL/min
Resolving Power	100	–
Analysis Duration	15	min

**Table 2:** Volatile organic compounds emitted by the three studied seaweeds.

Volatile Organic Compounds	*Grateloupia turuturu*	*Codium tomentosum*	*Bifurcaria bifurcata*
Ethanol	X	X	X
Diethyl Ether		X	X
Isopropanol	X		X
Acetone	X	X	X
Benzene	X		
2-Butanone	X	X	X
2-Ethylfuran	X	X	X
2-Pentanone	X		
Hexanal	X	X	X
2-Hexenal	X	X	X
4-Heptenal	X		
Heptanal	X		
2-Heptenal	X		

**Table 3:** Identification parameters of the detected volatile organic compounds.

Volatile Organic Compounds	Retention Time (s)	Relative Drift Time	CAS Number	Note
Ethanol	73	1.057	64-17-5	Monomer
		1.153		Dimer
Diethyl Ether	75	1.091	60-29-7	Monomer
		1.201		Dimer
Isopropanol	80	1.104	67-63-0	Monomer
		1.256		Dimer
Acetone	89	1.161	67-64-1	Monomer
Benzene	117	1.113	71-43-2	Monomer
2-Butanone	122	1.082	78-93-3	Monomer
		1.302		Dimer
2-Ethylfuran	133	1.081	3208-16-0	Monomer
2-Pentanone	170	1.146	107-87-9	Monomer
		1.439		Dimer
Hexanal	250	1.286	66-25-1	Monomer
		1.642		Dimer
2-Hexenal	368	1.216	6728-26-3	Monomer
		1.598		Dimer
4-Heptenal	394	1.191	6728-31-0	Monomer
Heptanal	405	1.366	111-71-7	Monomer
		1.789		Dimer
2-Heptenal	613	1.300	18829-55-5	Monomer
		1.783		Dimer

## Data Availability

The data presented in this study are available upon reasonable request from the corresponding author. The data are not publicly available due to privacy reasons.

## References

[1] Mabeau S., Fleurence J. (1993). Seaweed in food products: Biochemical and nutritional aspects. Trends Food Sci. Technol..

[2] Ganesan A.R., Subramani K., Shanmugam M., Seedevi P., Park S., Alfarhan A.H., Rajagopal R., Balasubramanian B. (2020). A comparison of nutritional value of underexploited edible seaweeds with recommended dietary allowances. J. King Saud Univ. Sci..

[3] Mouritsen O.G. (2013). Seaweeds: Edible, Available & Sustainable.

[4] Fleurence J., Levine I. (2016). Seaweed in Health and Disease Prevention.

[5] Kim S.K. (2011). Handbook of Marine Macroalgae: Biotechnology and Applied Phycology.

[6] Holdt S.L. (2011). Bioactive compounds in seaweed: Functional food applications and legislation. J. Appl. Phycol..

[7] Polat S., Trif M., Rusu A., Simat V., Cagalj M., Alak G., Meral R., Özogul Y., Polat A., Özogul F. (2021). Recent advances in industrial applications of seaweeds. Crit. Rev. Food Sci. Nutr..

[8] Salehi B., Sharifi-Rad J., Seca A.M., Pinto D.C.G.A., Michalak I., Trincone A., Mishra A.P., Nigam M., Zam W., Martins N. (2019). Current Trends on Seaweeds: Looking at Chemical Composition, Phytopharmacology, and Cosmetic Applications. Molecules.

[9] Vieira E.F., Soares C., Machado S., Correia M., Ramalhosa M.J., Oliva-teles M.T., Carvalho A.P., Domingues V.F., Antunes F., Oliveira T.A.C. (2018). Seaweeds from the Portuguese coast as a source of proteinaceous material: Total and free amino acid composition profile. Food Chem..

[10] Mouritsen O.G., Williams L., Bjerregaard R., Duelund L. (2012). Seaweeds for Umami Flavour in the New Nordic Cuisine. Flavour.

[11] Maarse H. (1991). Volatile Compounds in Foods and Beverages.

[12] Berneira L.M., Silva C.C., Passos L.F., Mansilla A., Aurélio M., Santos Z., Pereira C.M.P. (2021). Evaluation of volatile organic compounds in brown and red sub-Antartic macroalgae. Braz. J. Bot..

[13] Hosoglu M.I. (2017). Aroma characterization of five microalgae species using solid-phase microextraction and gas chromatography–mass spectrometry/olfactometry. Food Chem..

[14] Kawai T., Fujimura T., Haard N.F., Simpson B.K. (2000). Enzymes and Seaweed Flavor. Seafood Enzymes: Utilization and Influence on Postharvest Seafood Quality.

[15] Kladi M., Vagias C., Roussis V. (2004). Volatile halogenated metabolites from marine red algae. Phytochem. Rev..

[16] Mirzayeva A., Castro R., Barroso C.G., Durán-Guerrero E. (2021). Characterization and differentiation of seaweeds on the basis of their volatile composition. Food Chem..

[17] Wang P., Chen J., Chen L., Shi L., Liu H. (2021). Characteristic Volatile Composition of Seven Seaweeds from the Yellow Sea of China. Mar. Drugs.

[18] Armenta S., Alcala M., Blanco M. (2011). A review of recent, unconventional applications of ion mobility spectrometry (IMS). Anal. Chim. Acta.

[19] Wang S., Chen H., Sun B. (2020). Recent progress in food flavor analysis using gas chromatography-ion mobility spectrometry (GC-IMS). Food Chem..

[20] Karpas Z. (2013). Applications of ion mobility spectrometry (IMS) in the field of foodomics. Food Res. Int..

[21] Chen T., Chen X., Lu D., Chen B. (2018). Detection of Adulteration in Canola Oil by Using GC-IMS and Chemometric Analysis. Int. J. Anal. Chem..

[22] Espalha C., Fernandes J.M., Diniz M.S., Vassilenko V. Fast and Direct Detection of Biogenic Amines in Fish by GC-IMS Technology. Proceedings of the IEEE 6th Portuguese Meeting on Bioengineering—ENBENG.

[23] Moura P.C., Vassilenko V., Fernandes J.M., Santos P.H. Indoor and Outdoor Air Profiling with GC-IMS. Proceedings of the 11th Advanced Doctoral Conference on Computing, Electrical and Industrial Systems, DoCEIS 2020.

[24] Moura P.C., Vassilenko V., Ribeiro P.A. (2023). Ion Mobility Spectrometry Towards Environmental Volatile Organic Compounds Identification and Quantification: A Comparative Overview over Infrared Spectroscopy. Emiss. Control Sci. Technol..

[25] Scheinemann A., Sielemann S., Walter J., Doll T. (2012). Evaluation Strategies for Coupled GC-IMS Measurement including the Systematic Use of Parametrized ANN Training Data. Open J. Appl. Sci..

[26] Moura P.C., Vassilenko V. (2022). Gas Chromatography—Ion Mobility Spectrometry as a tool for quick detection of hazardous volatile organic compounds in indoor and ambient air: A university campus case study. Eur. J. Mass Spectrom..

[27] Moura P.C., Vassilenko V. (2023). Contemporary ion mobility spectrometry applications and future trends towards environmental, health and food research: A review. Int. J. Mass Spectrom..

[28] Gabelica V., Marklund E. (2018). Fundamentals of ion mobility spectrometry. Curr. Opin. Chem. Biol..

[29] Creaser C.S., Griffiths J.R., Bramwell C.J., Noreen S., Hill C.A., Thomas C.L.P. (2004). Ion mobility spectrometry: A review. Part 1. Structural analysis by mobility measurement. Analyst.

[30] Li F., Xie Z., Schmidt H., Sielemann S., Baumbach J.I. (2002). Ion mobility spectrometer for online monitoring of trace compounds. Spectrochim. B.

[31] Kanu B., Hill H.H. (2018). Ion mobility spectrometry detection for gas chromatography. J. Chromatogr. A.

[32] Fernades J.M., Vassilenko V., Moura P.C., Fetter V. Gas Chromatography—Ion Mobility Spectrometry Instrument for Medical Applications: A Calibration Protocol for ppb and ppt Concentration Range. Proceedings of the 12th Advanced Doctoral Conference on Computing, Electrical and Industrial Systems, DoCEIS 2021.

